# Ethanolic extract of the fungus *Trichoderma stromaticum* decreases inflammation and ameliorates experimental cerebral malaria in C57BL/6 mice

**DOI:** 10.1038/s41598-018-19840-x

**Published:** 2018-01-24

**Authors:** Yusmaris Cariaco, Wânia Rezende Lima, Romulo Sousa, Layane Alencar Costa Nascimento, Marisol Pallete Briceño, Wesley Luzetti Fotoran, Gerhard Wunderlich, Jane Lima dos Santos, Neide Maria Silva

**Affiliations:** 1 Laboratory of Immunopathology, Institute of Biomedical Sciences, Federal University of Uberlândia, Uberlândia, 38400-902 Minas Gerais Brazil; 2 Institute of Exact and Natural Sciences, Federal University of Mato Grosso, Rondonópolis, 78735-901 Mato Grosso Brazil; 3 Department of Parasitology, University of São Paulo, São Paulo, 05508-900 Brazil; 4 State University of Santa Cruz, Ilhéus, Bahia 45662-900 Brazil

## Abstract

Increased resistance to the first-line treatment against *P*. *falciparum* malaria, artemisinin-based combination therapies, has been reported. Here, we tested the effect of crude ethanolic extract of the fungus *Trichoderma stromaticum* (Ext-Ts) on the growth of *P*. *falciparum* NF54 in infected human red blood cells (ihRBCs) and its anti-malarial and anti-inflammatory properties in a mouse model of experimental cerebral malaria. For this purpose, ihRBCs were treated with Ext-Ts and analysed for parasitaemia; C57BL/6 mice were infected with *P*. *berghei* ANKA (PbA), treated daily with Ext-Ts, and clinical, biochemical, histological and immunological features of the disease were monitored. It was observed that Ext-Ts presented a dose-dependent ability to control *P*. *falciparum* in ihRBCs. In addition, it was demonstrated that Ext-Ts treatment of PbA-infected mice was able to increase survival, prevent neurological signs and decrease parasitaemia at the beginning of infection. These effects were associated with systemically decreased levels of lipids and IFN-γ, ICAM-1, VCAM-1 and CCR5 cerebral expression, preserving blood brain barrier integrity and attenuating the inflammatory lesions in the brain, liver and lungs. These results suggest that Ext-Ts could be a source of immunomodulatory and antimalarial compounds that could improve the treatment of cerebral malaria.

## Introduction

Although malaria cases globally fell over the last 16 years, the WHO estimated that 216 million new cases and 445,000 deaths from malaria occurred worldwide in 2016^1^. Malaria can present as uncomplicated fever paroxysm, where the body temperature sharply rises, leading to a ‘hot stage’ with flushes and tachycardia followed by a sharp fall of body temperature, profuse sweats or diaphoresis and often symptomatic orthostatic hypotension and anaemia; however, when the treatment is delayed, severe forms of disease may occur, leading to cerebral, pulmonary or renal impairment^2^.

The infection produced by *P*. *berghei* ANKA (PbA) in a mouse model of experimental cerebral malaria (ECM) has many similarities to human cerebral malaria (HCM), and parasitised red blood cell (pRBC)-dependent occlusion of brain capillaries and haemostasis are associated features of the disease in both HCM and ECM^3,4^. The high expression of pro-inflammatory cytokines, chemokines and adhesion molecules that are induced during the infection are associated with the onset of cerebral malaria (reviewed in ref.^5^). The expression of intercellular adhesion molecule-1 (ICAM-1) is upregulated on the cerebral vasculature endothelium of humans during malaria infection, and it is one of the major receptors involved in the sequestration of infected *P*. *falciparum* red blood cells within the brain, which is described in cerebral malaria (CM)^6^. The pathogenesis of ECM is correlated with TNF-α, which is associated with upregulation of ICAM-1 in the brain^7^. IFN-γ is also involved in ECM^8^ since mice lacking the α-chain of the IFN-γ receptor are resistant to ECM and lack any increases of TNF or ICAM-1 protein in the brain^9^. In addition, ICAM-1, VCAM-1 and P-selectin are all upregulated on the endothelium of the brain of mice susceptible to ECM (reviewed in ref.^10^). CC chemokine receptor 5 (CCR5) is also upregulated in the brain of infected individuals^11^, and its deficiency in the animal model protects against ECM by decreasing the amount of CCR5^+^ CD8^+^ T cells as well as reducing pro-inflammatory cytokine production^12^. In PbA ECM, the migration and the intracellular sequestration of leukocytes in the brain are also involved in the pathogenesis^13^. Both CD4^+^ and CD8^+^ T cells are required for development of ECM^14^, and the migration of cells is controlled by IFN-γ, mainly CD8^+^ T cell^13^. Comparable recruitment and activation of CD8^+^ T cells are found in the brains of mice infected with *P*. *berghei* NK65 (non-ECM-causing) or PbA (ECM-causing). However, perivascular brain-infiltrating CD8^+^ T cells display an arrested phenotype in PbA-infected mice^15^.

The treatment of severe malaria is based on a scheme of parenteral therapy followed by oral therapy with artemisinin-based combination therapies ACTs^16^. Although the use of artemisinin derivatives has succeeded at reducing the number of deaths from *falciparum* malaria in recent years^1^, the principal concern about the use of ACTs is the appearance of strains resistant to artemisinin derivatives reported in Southeast Asia^17,18^. Given the emerging resistance of parasites to ACTs, new therapies against malaria parasites are necessary to treat this disease. Anti-malarial drugs indirectly influence the immune response by means of their ability to destroy malaria parasites and thereby reduce the amount of antigens capable of activating the immune system. Additionally, some anti-plasmodial drugs, such as quinine, chloroquine and mefloquine, also exert a direct effect on the immune system by decreasing the production of pro-inflammatory cytokines such as TNF and IL-2, which are involved in the pathogenesis of severe malaria (reviewed by ref.^19^). In this sense, studies of immunomodulators for treatment of cerebral malaria as an adjunct therapy to control pro-inflammatory cytokine production and diminish the expression of adhesion molecules regulating parasite sequestration could improve the survival of individuals (reviewed by ref.^20^).

Fungi are natural producers of a broad range of secondary metabolites, some of which have shown antimicrobial potential^21^. Fungal secondary metabolites, such as penicillin, cephalosporins, statins, cyclosporin, mycophenolic acid, compactin and gliotoxin, have had diverse uses in clinical practice (reviewed by Kuck *et al*.)^22^. In this regard, some species of the genus *Trichoderma* have gained relevance in recent decades since they exhibit potent mycoparasitic properties. Additionally, they are also a source of volatile and non-volatile compounds that present antimicrobial properties against important phytopathogens^23^. *T*. *stromaticum* is a filamentous fungus that became important after being isolated from witches’ broom disease of cacao plantations in north-eastern Brazil. Subsequent studies demonstrated that *T*. *stromaticum* has a beneficial effect by inhibiting the development of fruiting bodies of *Crinipellis perniciosa*, a fungal pathogen of cacao^24,25^. Actually, a commercial product based on large-scale production of *T*. *stromaticum* spores (Tricovab®) has had rapid acceptance among cocoa farmers, since this fungus is pathogenic for neither humans, animals nor plants^26^. Additionally, some antibiotic peptides that might contribute to plant protection have been identified among their secondary metabolites^27^.

During the constant search for new anti-malarial agents, a number of fungal extracts have been tested as a potential drug against the parasite^28–30.^ Nevertheless, the effect of extracts of *T*. *stromaticum* on experimental malaria has not been tested yet. We have previously shown that the spores of the fungus *T*. *stromaticum* decrease IFN-γ and IL-10 production in Concanavalin A-stimulated spleen cells from BALB/c mice^31^. In the present study, we investigated the effects of the administration of crude ethanolic extract of *T*. *stromaticum* (Ext-Ts) in an ECM animal model. Clinical, histological, immunological and biochemical parameters of mice experimentally infected with PbA were assessed, and the protective effect of the Ext-Ts was determined.

## Results

### Ext-Ts treatment improves the survival rate and prevents neurological signs in C57BL/6 mice infected with PbA

**Figure 1:**
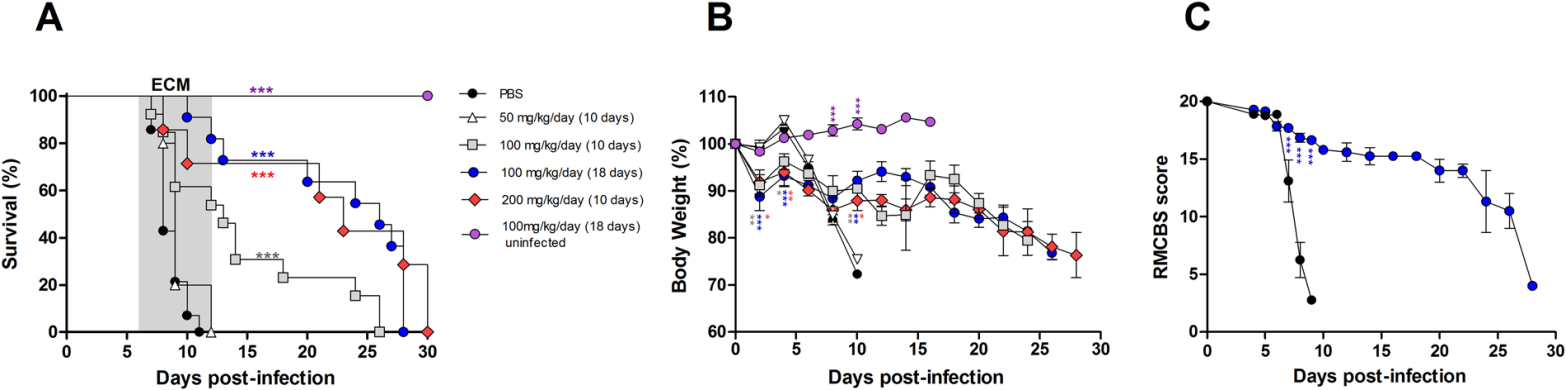
Administration of Ext-Ts increases the survival rate and decreases clinical signs associated with experimental cerebral malaria during murine PbA infection. Survival, body weight and RMCBS score of C57BL/6 mice treated with indicated concentrations of Ext-Ts four hours after inoculation of animals with 5 × 10^4^ red blood cells infected with PbA, and daily for 10 days or 18 days. Control mice were infected and treated with vehicle (PBS). A group of uninfected animals was treated with 100 mg/kg/day of Ext-Ts for 18 days. The survival (**A**), body weight (**B**) and RMCBS score (**C**) were evaluated. Data are presented as mean of each group ± SEM and are representative of at least two independent experiments. The survival rate was analysed by Chi-square test; body weight and RMCBS score by Two-way ANOVA, followed by Bonferroni post-test. Statistical differences in relation to the PBS group are pointed according to the colour of the symbol of each group. *p < 0.05, **p < 0.01, ***p < 0.001.

To determine whether the extract could interfere in the immune response induced by *Plasmodium* and ameliorate ECM, C57BL/6 mice were inoculated with 5 × 10^4^ PbA-infected RBCs and treated with 50 mg/kg/day of Ext-Ts for 10 days, 100 mg/kg/day of Ext-Ts for 10 or 18 days or 200 mg/kg/day of Ext-Ts for 10 days. Untreated C57BL/6 control mice and animals treated with 50 mg/kg/day of Ext-Ts were highly susceptible to PbA inoculation, and the animals died until 10 days post-infection (d.p.i.). On the other hand, animals treated with 100 mg/kg Ext-Ts were significantly more resistant to infection in comparison with untreated and infected mice (p < 0.001), mainly when they were treated for 18 days (Fig. 1A). In parallel, animals treated with 200 mg/kg Ext-Ts for 10 days presented resistance to infection similar to that of mice treated with 100 mg/kg/day for 18 days.

Mice treated with Ext-Ts 100 or 200 mg presented significant weight loss during the first 4 days of infection and treatment compared with the untreated group (Fig. 1B). However, on day 10 p.i., untreated or 50 mg-treated C57BL/6 mice presented higher weight loss compared with 100 or 200 mg Ext-Ts-treated mice (Fig. 1B). Additionally, 100 or 200 mg Ext-Ts-treated animals were able to maintain constant weight for 10 additional days. Subsequently, a considerable reduction in weight was also detected in these groups of mice (Fig. 1B). Ext-Ts-treated uninfected mice increased their weight during the observation period (Fig. 1B). Thus, we decided to treat animals with 100 mg/kg/day for 18 days in additional experiments.

When testing for symptoms of ECM, the first parameters that changed in all PbA-infected animals were balance and motor performance. Untreated and infected mice developed ataxia and progressive paralysis, loss of limb strength and pinna reflex, ruffled fur and death from 7–9 d.p.i., reaching 2.5 on the Rapid Murine Coma and Behavior Scale score (RMCBS) (Fig. 1C). In contrast, in the same period, infected animals treated with Ext-Ts presented distinctly higher RMCBS values (p < 0.0001) (Fig. 1C). After 10 days p.i. Ext-Ts-treated mice managed to maintain a stable clinical score for an additional 15 days, with altered balance, motor performance and body position. In the later period of infection, surviving Ext-Ts-treated mice presented a decrease in RMCBS; however, the score was still above 10 on day 26 of infection (Fig. 1C). The uninfected Ext-Ts-treated animals did not present any alteration in mortality rate, weight loss or morbidity score.

### Ext-Ts treatment controls the parasitaemia of PbA-infected mice in the early period of infection

**Figure 2:**
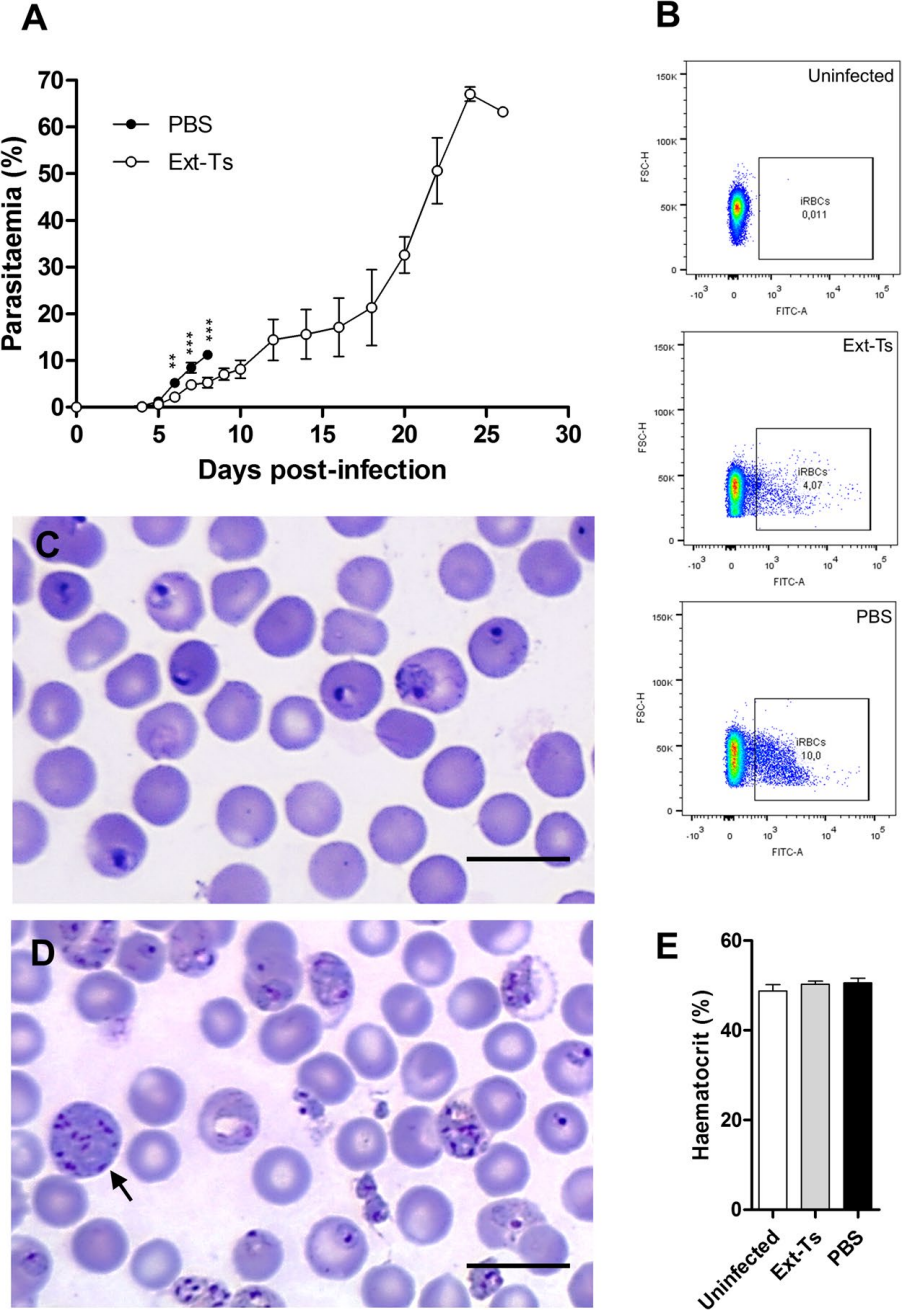
Ext-Ts was able to delay the increase of parasitaemia levels in C57BL/6 mice infected with PbA. Blood tail samples were collected from mice inoculated with 5 × 10^4^ infected red blood cells and treated with Ext-Ts 100 mg/kg (n = 10) or vehicle (PBS, n = 12) during 18 days for parasitaemia analysis through flow cytometry. (**A**) Time course of parasitaemia; (**B**) Representative dot plot graphs of parasitaemia on day 8 p.i. showing the frequency of iRBCs; (**C**) Peripheral-blood smear of untreated (PBS) and (**D**) Ext-Ts treated-mice on day 8 p.i., and (**E**) Haematocrit on day 7 p.i. The arrow point to schizont stage. Scale bar: 10 µm. Data are presented as mean of each group ± SEM at indicated time points and are representative of at least two independent experiments. **p < 0.01, ***p < 0.001.

During the course of treatment and infection, parasitaemia was estimated by flow cytometry. Infected and untreated animals developed a progressive increase in parasitaemia, and from day 6 to 8 after infection, parasitaemia of 5–11% was measured, coinciding with the death of the animals of this group on 9 d.p.i. Ext-Ts-treated mice also showed a gradual increase in parasitaemia levels during the course of infection. However, the average parasitaemia levels in Ext-Ts-treated mice were significantly lower from 6 to 8 d.p.i. (2–5%) (p < 0.01) compared with infected untreated mice (Fig. 2A). In accordance, the haematocrit of infected untreated and infected Ext-Ts-treated mice presented values similar to those of uninfected animals at 7 d.p.i. (Fig. 2E).

Between days 8 and 10 after infection, parasitaemia in the treated infected mice remained between 5 and 8%, after which it increased dramatically over the next 16 days (Fig. 2A), culminating with the death of the animals until day 28 p.i. The reduction in parasitaemia was noted in blood smears, where we also observed a decrease of advanced intra-erythrocytic parasite stages in red blood cells (RBCs) (Fig. 2C).

### Ext-Ts treatment decreases haemozoin deposition in mouse livers

**Figure 3:**
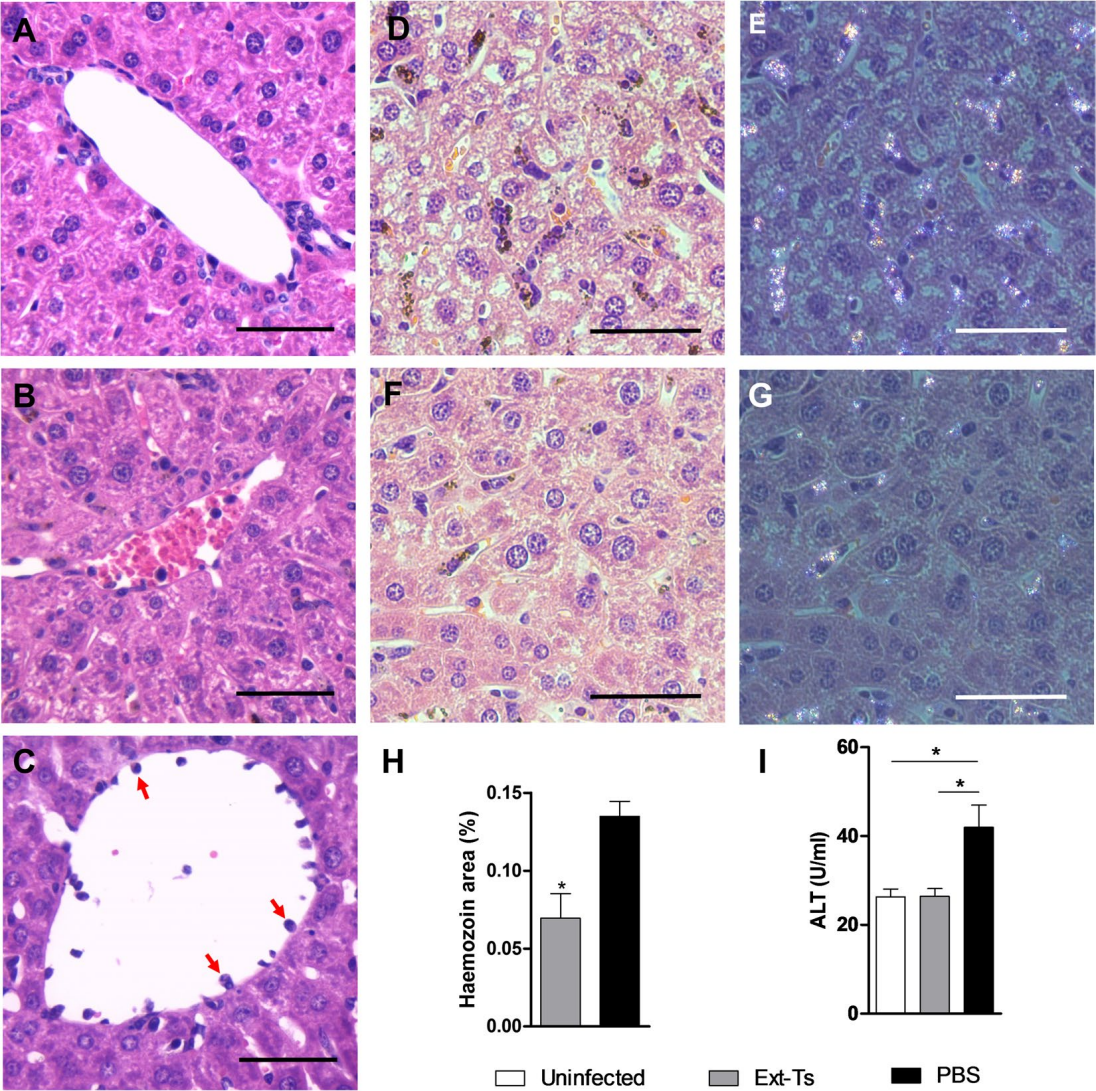
Ext-Ts treatment reduces haemozoin deposition in the liver of PbA-infected mice. C57BL/6 mice were inoculated with 5 × 10^4^ infected red blood cells and treated with Ext-Ts 100 mg/kg (n = 6) or vehicle (PBS, n = 5) and euthanized on day 7 p.i. An uninfected group (n = 5) was used as control. Haemozoin quantification was performed on images of liver tissue sections, stained with H&E and captured using polarization filter; the images were analysed by ImageJ. Histological section of liver stained with H&E of (**A**) uninfected, (**B**) infected Ext-Ts treated, and (**C**) infected untreated animals, showing leukocyte cyto-adherence to blood vessel endothelium (red arrows). Representative images of liver tissue sections captured by conventional light microscopy (**D** and **F**) and polarized light microscopy (**E** and **G**) where haemozoin is identified as brown and birefringent accumulation, respectively. (**D** and **E**) images were taken from an infected untreated animal. (**F** and **G**) images represent the liver section from an infected and Ext-Ts-treated animal. Scale bar: 50 µm. (**H**) Haemozoin amounts expressed as a percentage of the total area of the image (in pixels); (**I**) Serum levels of ALT. Data are presented as mean of each group ± SEM, and are representative of at least two independent experiments. *p < 0.05.

The liver is one of the main organs damaged by blood stage *Plasmodium* species, inducing jaundice, hepatomegaly and liver enzyme elevation^32,33^. Therefore, we investigated the protective effect of Ext-Ts in the liver under PbA infection. Histological analyses of uninfected animals were used as controls (Fig. 3A), showing that the liver of PbA-infected mice presented congested blood vessels (Fig. 3B) with prominent cyto-adherence of leukocytes (Fig. 3C). However, the Ext-Ts-treated and infected animals presented fewer severe lesions (despite the observation of congested blood vessels), fewer cyto-adhered leukocytes and lower numbers of haemorrhagic areas.

Haemozoin (Hz) is a malaric pigment released from ruptured iRBCs that can accumulate in tissues, where it is often internalised by circulating and resident macrophages. Alternatively, Hz in macrophages may stem from phagocytosed iRBCs. The deposition of this pigment appears to have deleterious effects on tissues^34,35^. Thus, we evaluated the amount of Hz accumulated in the liver of the trial groups using conventional light microscopy and microscopy with a polarised light filter (Fig. 3D–G). The images identified Hz in histological sections of liver from infected mice as a dark brown pigment in light microscopy (Fig. 3D,F) and as birefringent accumulations in polarised light microscopy (Fig. 3E,G). In the liver of untreated mice, Hz appeared as large aggregates (Fig. 3D,E). In contrast, in the liver of Ext-Ts-treated mice, the pigment was found to have lower intensity (Fig. 3F,G). Hz was quantified in the hepatic tissue by using ImageJ software in images captured by the polarised microscope. It was observed that infected Ext-Ts-treated mice presented lower birefringence in relation to total area compared with untreated infected mice (p < 0.05) (Fig. 3H). These data were consistent with the lower serum ALT levels in both uninfected and infected Ext-Ts-treated mice when compared with infected untreated mice (p < 0.05) (Fig. 3I). Thus, the reduced Hz deposition in the liver and ALT serum levels observed on day 7 p.i. may reflect the lower parasitaemia observed in Ext-Ts-treated infected mice.

### PbA-infected Ext-Ts-treated mice show less oedema and fewer haemorrhagic lesions in lungs

**Figure 4:**
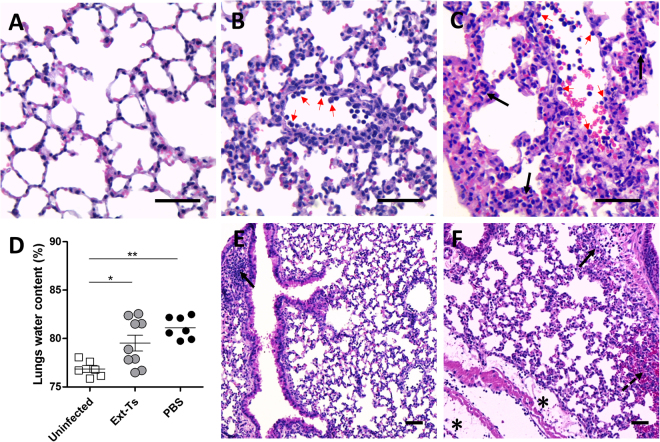
Administration of Ext-Ts decreases inflammation and oedema in the lung of PbA-infected mice. C57BL/6 mice were inoculated with 5 × 10^4^ red blood cells infected with PbA, treated daily with 100 mg/kg/day of Ext-Ts and euthanized on day 7 p.i. Histological section of lung of uninfected (**A**), infected and Ext-Ts-treated (**B**,**E**) and infected untreated mice (**C**,**F**) stained with H&E, showing inflammatory infiltrates (black arrows), oedema areas (black asterisk), haemorrhagic areas (dashed arrow) and cyto-adherence of leukocytes (red arrows). Scale bar: 50 µm; (**B**) Lungs water content of uninfected (n = 6), untreated (PBS, n = 7) and Ext-Ts-treated mice (Ext-Ts, n = 9). Data are presented as mean of each group ± SEM and are representative of at least two independent experiments. *p < 0.05, **p < 0.01.

Another landmark deleterious event in PbA infection is the occurrence of lung lesions and oedema^34^. The pulmonary lesions of PbA-infected mice were constituted by inflammatory infiltrate of mononuclear cells within the alveolar walls, enlarging the pulmonary septum in comparison with uninfected tissue (Fig. 4A,B and E). Additionally, cyto-adherence of leukocytes was detected in the epithelia of the blood vessels (Fig. 4B,C), and some haemorrhagic areas were also detected in infected animals (Fig. 4F). In Ext-Ts-treated mice, milder inflammatory infiltrate in the pulmonary septum was observed, with less oedema and fewer haemorrhagic areas.

Given that cerebral and pulmonary oedema are common features of severe malaria due to increased capillary permeability that leads to intravascular fluid loss^34,36^, we evaluated pulmonary oedema in the lungs. Accordingly, there were extensive areas of oedema surrounding some blood vessels (Fig. 4F, highlighted by asterisks) and bronchi of infected untreated animals. Pulmonary oedema was also analysed based on the measurement of lung water content estimated by the wet-dry method. It was observed that infected Ext-Ts-treated and infected untreated mice presented statistically higher lung water content compared with uninfected mice (p < 0.05, p < 0.01, respectively) (Fig. 4D). Although not statistically significant, infected Ext-Ts-treated mice presented a tendency to have lower water content in the lungs compared with untreated infected mice (Fig. 4D). These results suggest that administration of Ext-Ts was able to reduce but not prevent oedema production into the lung tissue of PbA-infected mice.

### Ext-Ts treatment preserves blood brain barrier integrity and diminishes cerebral inflammation in PbA-infected mice

**Figure 5:**
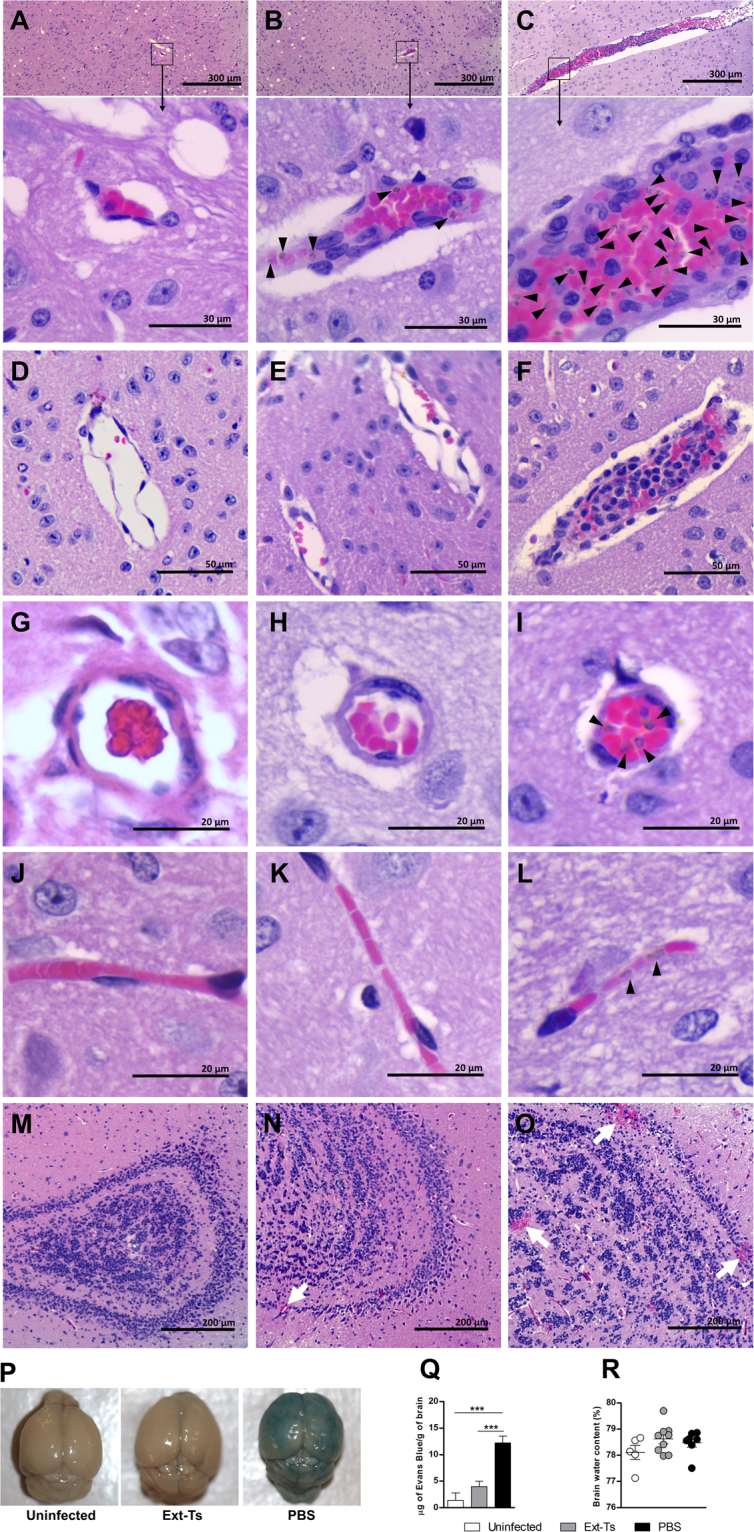
Ext-Ts treatment decreases histological alterations into the brain tissue and blood-brain barrier leakage on a murine model of experimental cerebral malaria. C57BL/6 mice were inoculated with 5 × 10^4^ red blood cells infected with PbA, treated daily (n = 9) or not (n = 7) with 100 mg/kg/day of Ext-Ts. After 7 days of infection, a solution of 2% Evans blue dye was injected intraperitoneally; two hours later, transcardially perfusion was performed with saline and the brain was collected, weighted and homogenised in Na_2_SO_4_/Acetone overnight. Absorbance was measured and Evans blue dye amounts estimated from a standard curve. An uninfected untreated group was used as negative control (n = 5). Histological sections of brain of uninfected (**A**,**D**,**G**,**J**,**M**), infected Ext-Ts-treated (**B**,**E**,**H**,**K**,**N**) and infected untreated mice (**C**,**F**,**I**,**L**,**O**) stained with H&E. Squares and black arrows point the regions in the brain that are amplified in A–C, arrowheads point infected red blood cells within the blood vessels of infected animals and white arrows point haemorrhagic areas in olfactory bulb (**N**,**O**); (**P**) Macroscopic image of the brain showing Evans blue dye leakage in cerebral tissue of studied groups; (**Q**) Evans blue dye quantification in cerebral tissue; (**R**) Brain water content. Data are presented as mean of each group ± SEM and are representative of at least two independent experiments. ***p < 0.001.

Analysis of histological brain sections of infected untreated mice (Fig. 5C,F,I,L) showed the presence of a prominent intravascular accumulation of iRBCs and leukocytes in the lumen of most cerebral blood vessels at 7 d.p.i. (Fig. 5C,I), some of them enriched with parasitic material. iRBCs were also found within apparently obstructed capillaries (Fig. 5L). In addition, several haemorrhagic areas were detected in the cerebral parenchyma of infected untreated mice (Fig. 5O). In Ext-Ts-treated mice, the lesions were less severe (Fig. 5B,E,H,K), with lower numbers of RBCs and leukocytes congesting blood vessels (Fig. 5B) compared with infected untreated mice (Fig. 5C). Additionally, lower iRBC numbers (Fig. 5B) and fewer haemorrhagic areas (Fig. 5N) were detected in the brains of Ext-Ts-treated mice. The permeability of the blood-brain barrier (BBB) was also analysed through quantification of Evans blue dye leakage. As expected, uninfected mice showed no disruption of the BBB (p < 0.001) (Fig. 5P,Q). In contrast, untreated PbA-infected mice presented prominent dye accumulation in cerebral tissue, indicating a breakdown of the BBB. Infected mice that received Ext-Ts treatment displayed less staining of the brain by Evans blue (Fig. 5P). Evans blue dye leakage was significantly lower in infected Ext-Ts-treated mice in comparison with infected untreated mice (p < 0.001) (Fig. 5P,Q). The water content in the brain was also estimated as a measurement of fluid extravasation into the tissue. The brain water content was similar between uninfected and PbA-infected mice, irrespective of whether they were treated with Ext-Ts (Fig. 5R).

### The IFN-γ, ICAM-1 and CCR5 mRNA expression levels are reduced in the brain of PbA-infected Ext-Ts-treated mice

**Figure 6:**
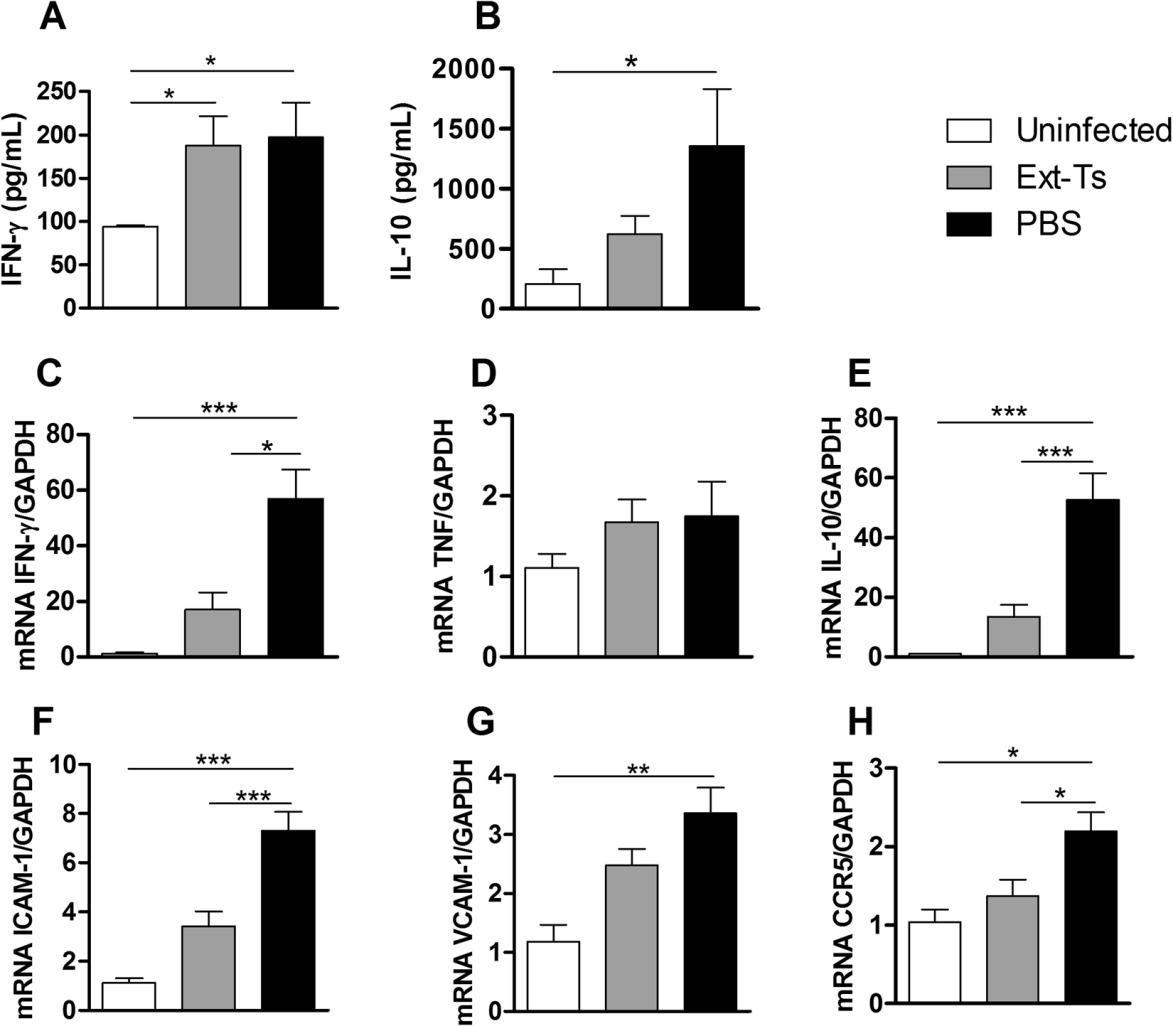
Ext-Ts treatment interferes in IFN-γ, IL-10, ICAM-1, VCAM-1 and CCR5 mRNA expression in the brain of PbA-infected mice. C57BL/6 mice were inoculated with 5 × 10^4^ red blood cells infected with PbA, treated daily with 100 mg/kg/day of Ext-Ts and euthanized on day 7 p.i. Levels of IFN-γ (**A**) and IL-10 (**B**) in serum samples were measured by ELISA. IFN-γ (**C**), TNF (**D**), IL-10 (**E**), ICAM-1 (**F**), VCAM-1 (**G**) and CCR5 (**H**) transcripts expression in cerebral tissue were quantified by qPCR on day 7 p.i. Results are presented as mRNA expression of untreated or Ext-Ts-treated PbA-infected mice, relative to uninfected mice. The relative levels of gene expression were calculated by reference to the GAPDH in each sample, using the threshold cycle (Ct) method, and GAPDH levels were similar in all samples. Data are presented as mean of each group ± SEM and are representative of at least two independent experiments. *p < 0.05, **p < 0.01, ***p < 0.001.

To elucidate the role of Ext-Ts treatment in protection from ECM, cytokine levels were measured systemically as well as the mRNA expression of cytokines, adhesion molecules and the CCR5 chemokine receptor in brain tissue. Higher IFN-γ levels were found in serum of infected animals whether or not they were Ext-Ts-treated (p < 0.05) in comparison with uninfected mice (Fig. 6A). IL-10 levels were increased during infection; these levels were significantly higher in infected untreated mice than in non-infected mice (Fig. 6B), and Ext-Ts reduced the cytokine levels relative to PbA-infected mice, although not statistically significantly (Fig. 6B). In the brain, the infection increased IFN-γ expression in untreated mice (p < 0.001), and to a less extension in Ext-Ts-treated mice, which was lower in comparison with untreated infected mice (p < 0.05) (Fig. 6C). TNF expression levels were not altered in the brain tissues of infected mice, although these values tended to be higher in infected animals (Fig. 6D). The quantity of IL-10 transcripts was higher in infected untreated mice when compared with uninfected (p < 0.001) or infected Ext-Ts-treated mice (p < 0.001) (Fig. 6E).

The mRNA expression of ICAM-1 was elevated in infected untreated mice when compared with uninfected (p < 0.001) or infected Ext-Ts-treated mice (p < 0.001) (Fig. 6F). In addition, the VCAM-1 expression levels were higher in infected untreated mice in comparison with uninfected animals (p < 0.01) (Fig. 6G). Although lacking statistical significance, the Ext-Ts-treated mice showed higher VCAM-1 expression compared with uninfected animals and lower in relation to infected untreated mice (Fig. 6G). Regarding CCR5, it was observed that infected untreated animals presented higher mRNA expression of this chemokine receptor in the brain compared with uninfected (p < 0.05) or infected Ext-Ts-treated mice (p < 0.05) (Fig. 6H). Interestingly, the infected Ext-Ts-treated mice presented CCR5 expression similar to that of uninfected mice (Fig. 6H).

### Cholesterol and triglyceride serum concentrations are diminished by Ext-Ts treatment of PbA-infected mice

**Figure 7:**
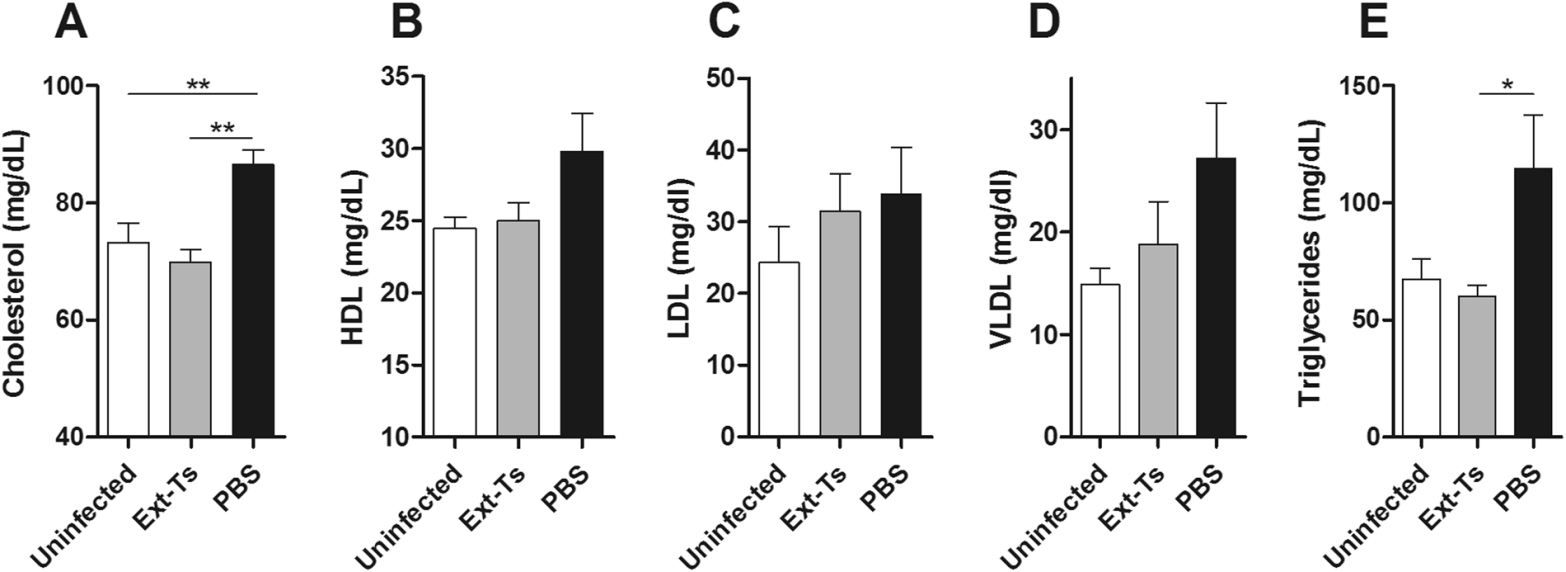
Serum lipids are modulated in PbA-infected mice treated with Ext-Ts. C57BL/6 mice were inoculated with 5 × 10^4^ red blood cells infected with *P*. *berghei* ANKA, treated daily with 100 mg/kg/day of Ext-Ts or vehicle (PBS) and euthanized on day 7 p.i. Uninfected animals were used as control. Serum concentrations of total cholesterol (**A**), HDL (**B**), LDL (**C**), VLDL (**D**) and triglycerides (**E**) were measured (n = 5/group). Data are presented as mean of each group ± SEM and are representative of at least two independent experiments. *p < 0.05, **p < 0.01.

Since parasites need a constant supply of lipids from the host for their development^37^, we decided to evaluate the lipid profile in the studied groups. Ext-Ts treatment reduced the elevated cholesterol and triglyceride levels that were found in infected untreated mice but did not affect the elevated lipoprotein content (Fig. 7A–E).

### Growth inhibition assays using Ext-Ts-treated parasites

**Figure 8:**
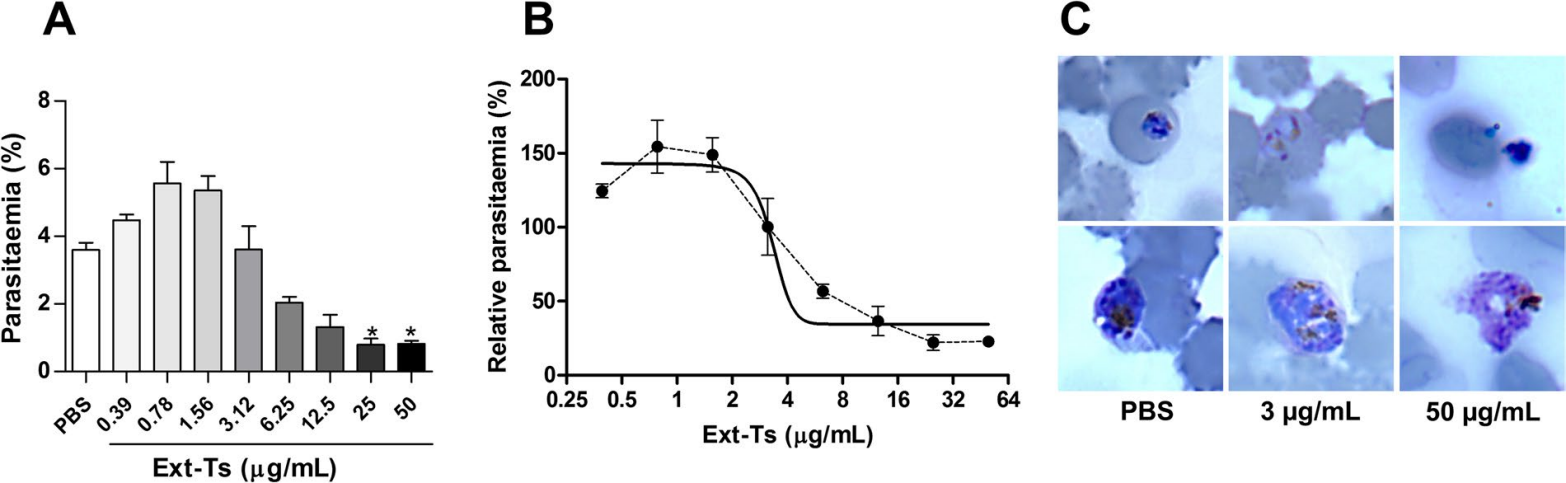
*Trichoderma stromaticum* extract (Ext-Ts) leads to enhanced parasite growth of *Plasmodium falciparum* at low concentrations but to dose-dependent inhibition in higher doses. *P*. *falciparum* NF54 parasites were incubated for 48 h at the given Ext-Ts dilutions in a total volume of 200 µL. The final parasitaemias were then measured by flow cytometry using Ethidium bromide staining as described. A dose-response curve was fitted (solid line) to the data points (dotted line). (**A**,**B**) Curve dose-response, (**C**) Representative images of parasites morphology after 48 hours of indicated treatments in Giemsa-stained blood smears. Data are presented as mean ± SEM and are representative of an experiment performed in triplicate. *p < 0.05.

In order to check the effect of the PBS-soluble fraction of Ext-Ts on *in vitro* cultures of *P*. *falciparum*, cultures were incubated for 48 h with increasing concentrations of Ext-Ts. As shown in Fig. 8A and B, a bimodal effect was observed. While lower concentrations led to an enhancement of parasite growth, extract dilutions above 3–4 µg/ml resulted in a dose-dependent inhibition and stalled parasite development/parasite death at the highest concentration of 50 µg/ml (Fig. 8C). The estimated EC_50_ for the extract was 5.2 μg/mL.

## Discussion

Fungi are rich in a variety of secondary metabolites because, similar to plants, these constitute their principal defence mechanism against predators. The genus *Trichoderma* is a strong producer of secondary metabolites with antimicrobial activity (reviewed by ref.^38^). Many of these molecules are the so-called peptaibiotics, defined as linear peptide antibiotics characterised by the presence of α-amino-isobutyric acid (Aib). The majority of Aib-containing peptides carry a C-terminal residue representing a 2-amino alcohol; this subgroup is referred to as a ‘peptaibol’. Specifically, *T*. *stromaticum*, which belongs to the order Hypocreales, presents a peptaibol named trichostromaticin that contributes to the potent bioactivity of the fungus against plant pathogens^27^. Recently, it was demonstrated that *Trichoderma viride* mycelium has nematophagous activity, which reduces the infectivity of *Toxocara canis* eggs^39^. From *Trichoderma longibrachiatum*, peptaibols have been identified such as Trichogin GA IV, a lipopeptaibol that shows strong activity against *Staphylococcus aureus* and other Gram-positive bacteria^40^. In this regard, other peptaibols named longibrachins were also identified in *Trichoderma longibrachiatum*, and these compounds present antifungal and antibacterial properties, in addition to acute toxicity on Dipteran larvae^41^.

Herein, we tested the extract of the fungus *T*. *stromaticum* in a mouse model of inflammation induced by PbA that leads to ECM in C57BL/6 mice. It was previously demonstrated that *T*. *stromaticum* spores downregulate the respiratory burst and nitric oxide production in mouse phagocytes, and intranasal sensitisation of BALB/c mice with spores decreased IL-10 production in the bronchoalveolar fluid and IFN-γ and IL-10 production by spleen cells *in vitro*^31^. In the present investigation, it was demonstrated that the fungal extract can also affect the immune response against *P*. *berghei* infection, since Ext-Ts administration had a protective effect during infection by PbA in C57BL/6 mice, infected Ext-Ts-treated animals showing prolonged survival as well as a decrease of neurological alterations associated with ECM, resembling resistant mice.

Anaemia is a remarkable symptom during malaria, although its presentation in humans is variable, depending on age, duration of disease and haemoglobin levels at the onset of malaria^2^. In this work, no significant difference was found in haematocrit of the experimental groups at 7 d.p.i. irrespective of whether they were treated with Ext-Ts, as haematocrit strictly depends on parasitaemia, and in this period, the parasitaemia of untreated mice reached a maximum of 9%. Here, we reported a notable decrease in parasitaemia levels in the first 8 days of infection in PbA-infected mice treated with Ext-Ts. In accordance, previous research found that C57BL/6 mice inoculated with 1 × 10^5^ PbA-infected RBCs and treated with aqueous extract of the fungus *Agaricus blazei* also showed reduced parasitaemia and enhanced survival, this effect being associated with diminished expression of IL-1β, TNF and IL-6 in the brain^29^.

During erythrocytic stages, *Plasmodium* invades RBCs, digests haemoglobin through the degradation of globin into amino acids needed for its metabolism and releases haem, which is toxic to the parasite itself. Because of this, the parasite performs dimerisation and crystallisation of haem into a harmless pigment, haemozoin^42^. After parasite maturation to schizont stages, iRBC rupture occurs and haemozoin is released and reaches different organs, mainly the liver, where this pigment accumulates, triggering local inflammation^35^. In this sense, a positive correlation between haemozoin deposition in the liver and parasitaemia levels has been demonstrated, and haemozoin gradually increases during infection^35^. In concordance, our data revealed elevated levels of haemozoin deposition in the liver of infected mice; however, treatment of infected mice with Ext-Ts was able to diminish the pigment deposition in the liver as well as decrease peripheral parasitaemia. In parallel, reduced hepatic tissue injury, such as decreased cyto-adherence and inflammatory infiltrate in the parenchyma, was observed in Ext-Ts-treated mice, revealing a protective effect of Ext-Ts. Consistent with the lower inflammatory alterations demonstrated in our experimental work, lower levels of ALT in infected Ext-Ts-treated mice were detected since ALT is a known marker of hepatic damage^43^.

In a systematic review and meta-analysis, it was demonstrated that among 42 studies, 83% reported hypocholesterolaemia, 87% a large decrease in HDL-c, 81% lower LDL-c, and 78% hypertriglyceridemia concentrations in patients with malaria^44^. In our investigation, we observed increases in cholesterol and triglyceride levels in serum samples of C57BL/6 mice infected with PbA presenting symptoms of ECM. In accordance, it was demonstrated that in C57BL/6 mice infected with PbA, serum triglycerides, VLDL and total cholesterol concentrations were enhanced in mice with ECM compared with mice that did not develop ECM^45^. In the study of Visser *et al*.^44^ it was not specified if the patients were suffering from CM. Despite the ability to synthetise fatty acids de novo, the parasite depends on a constant supply of host lipids, since the inhibition of host cholesterol sources leads to a decrease in the cholesterol content of hepatic merozoites^46^. Thus, the non-altered cholesterol and triglycerides in serum samples of Ext-Ts-treated mice 7 d.p.i. observed in our experimental work could also reflect the effect of Ext-Ts on parasite development. In concordance with our results, Sriwiphat *et al*.^47^ reported significant increases in total cholesterol and triglyceride levels in plasma of PbA-infected ICR mice, and treatment with crude aqueous extract of the medicinal plant *Andrographis paniculata* at 300 and 600 mg/kg significantly reduced the total cholesterol and triglycerides. Oluba *et al*.^30^ also associated the anti-malarial effect of an aqueous extract of *G*. *lucidum* with modulation of serum and liver lipids. It is also possible that variation in the serum lipid profile during malaria has a correlation with anaemia. Triglycerides and VLDL were elevated, whereas LDL levels were lower in adult Indian patients with iron deficiency anaemia from BRIMS, Bidar^48^. Although in the present work, anaemia was not detected at 7 d.p.i., we do not rule out that serum lipid alterations could be associated with anaemia in posterior periods of infection in Ext-Ts-treated infected mice.

Pulmonary and cerebral oedema are often produced during malaria infection^34,36^. In the lungs, this fluid infiltration from alveolar capillaries towards the interstitial space has been associated with sequestration, microvascular obstruction, increased capillary permeability and a reduction in oncotic pressure^49^. Additionally, ultrastructure studies showed that lung pathology during malaria has been associated with thickening of the basal membrane of the pulmonary endothelium and the congestion of alveolar capillarity with infected and uninfected RBCs^50^. Herein, pulmonary oedema was observed in infected mice irrespective of treatment with Ext-Ts.

There is a correlation between the breakdown of the blood brain barrier and cerebral malaria pathogenesis in humans and in a mouse experimental model (reviewed by refs^8,51^). Cerebral oedema resulting from enhanced BBB permeability is a common feature of HCM^52^ and ECM^4^. In the present investigation, despite no evident cerebral oedema detected on day 7 p.i., a breakdown of the BBB was observed in PbA-infected mice. Interestingly, Ext-Ts-treated PbA-infected mice maintained BBB integrity, which is in agreement with the lower intensity of cerebral lesions compared with infected animals.

Previous studies have shown that IFN-γ is essential for the development of experimental CM^9,14,53,54^, and it has been proposed that its action is through the induction of TNF^9^. TNF^7,53^ and lymphotoxin α (LT-α), a related member of the TNF family^55^, in ECM stimulate ICAM-1 expression in brain endothelial cells, leading to leukocyte sequestration in the brain^9,56^. In the present investigation, it was observed that PbA infection increased IFN-γ levels systemically and its expression in the brain, and Ext-Ts-treatment of infected mice was able to diminish the IFN-γ expression locally but not in serum samples. The TNF expression levels in the cerebral tissue were also verified; however, no significant increase was observed in its levels in infected compared to uninfected animals. In accordance with our study, other authors could also not find any increase in TNF message in the brain of 129 Sv/Ev × C57BL/6 mice at 6 d.p.i. with 10^6^ PbA iRBCs^7^. It was previously demonstrated that 129 Sv/Ev mice infected with 10^6^ iRBCs showed an increase in TNF-α levels in serum of IFN-γR ko and WT mice with ECM, and amounts of bioactive TNF-α in the brain of WT mice with and without ECM^9^. 129 Sv/Ev × C57BL/6^54^ and BALB/c^57^ mice infected with 10^6^ PbA iRBCs presented an increase in TNF-α levels in serum samples at 7 d.p.i. and 6 d.p.i., respectively. The different results for local TNF levels detected in our experimental investigation could be related to the different parasite burden used to infect the animals. Additionally, the role of TNF in the development of ECM is controversial, since it was previously shown that LTα, which binds both TNFR1 and 2, but not TNF-α, is the principal mediator of murine CM^55^. Regarding IL-10, infection of C57BL/6 mice with PbA leads to increased levels of IL-10 in serum samples and IL-10 transcripts in cerebral tissues. The extract treatment of infected mice decreased the levels of IL-10 in serum and its transcripts in the brain, despite these levels being higher compared with uninfected mice. In the absence of IL-10, 129 × C57BL/6 mice developed cerebral malaria when infected with 10^6^ PbA iRBCs^14^. In agreement with the present experimental work, C57BL/6 J × 129/Ola mice infected with 10^6^ PbA iRBCs presented significantly elevated levels of IL-10 under infection, mainly in those not suffering CM^12^; and 129 Sv/Ev mice infected with 10^6^ or 5 × 10^5^ PbA iRBCs showed IL-10 mRNA expression in the brain; however, they developed ECM at 6–9 d.p.i.^9^. Additionally, IL-10 levels gradually increased in serum with the progression of infection of BALB/c mice with 10^6^ PbA iRBCs, and neutralisation of IL-10 activity leads to symptoms of CM, although not as severe as in infected C57BL/6 mice^57^. Thus, increased IL-10 levels during infection of mice with PbA seem to be important for counter-regulating the pro-inflammatory cytokines and ameliorating ECM. In our investigation, the decreased IL-10 mRNA expression levels in the brain tissue of infected Ext-Ts-treated mice could be related to the lower IFN-γ expression compared to infected non-treated mice.

Histopathological study of the brains of mice with CM shows that vessels are generally distended, with the lumen packed with adhering iRBCs and leukocytes and with areas of haemorrhage. iRBCs in close contact with the endothelium and strong ICAM-1 staining were observed in capillaries and post-capillary venules during ECM^58^. TNF and LT-α induce strong and moderate VCAM-1 mRNA and protein expression, respectively, in a mouse brain endothelial cell line (b-end cells), and IFN-γ synergises with LT-α to enhance these expression levels. In addition, TNF, LT-α and IFN-γ induce strong, modest and slight ICAM-1 mRNA expression in b-end cells, respectively. However, when IFN-γ was added together with either TNF or LT-α there was a very strong synergistic effect^59^. Additionally, ICAM-1 is upregulated in brain endothelial vessels of mice with ECM in a TNF-dependent manner^7^. The present study showed that the infection with PbA increased ICAM-1 and VCAM-1 mRNA expression in the brain of infected mice. In accordance, C57BL/6 mice on day 6 p.i. with PbA presented high ICAM-1, VCAM-1 and P-selectin protein expression in the brain and lungs; and IFN-γ deficiency decreased ICAM-1 expression but not TNF deficiency^60^. Moreover, therapy with anti-adhesion molecule LFA-1 and VLA-4, ligands for ICAM-1 and VCAM-1, respectively, prevented fatal ECM by rapidly displacing luminal CD8^+^ T cells from cerebrovascular endothelial cells^61^. Interestingly, we demonstrated that the Ext-Ts treatment was able to reduce the mRNA expression of ICAM-1, and to a lower extent that of VCAM-1, in the brain of infected mice in comparison with infected untreated mice. In parallel, less severe lesions were observed in the brain of infected Ext-Ts-treated mice, with some RBCs and leukocytes congesting blood vessels, but only rarely leukocyte cyto-adherence. Children who had died of CM presented increased mRNA and protein expression levels of CCR5 in cerebral tissue^11^. In accordance, mice that are genetically deficient in CCR5 are resistant to ECM^12^. Interestingly, in the present experimental work, it was observed that treatment with Ext-Ts was able to diminish the expression of CCR5 in the brain of infected animals when compared with untreated infected mice.

**Figure 9:**
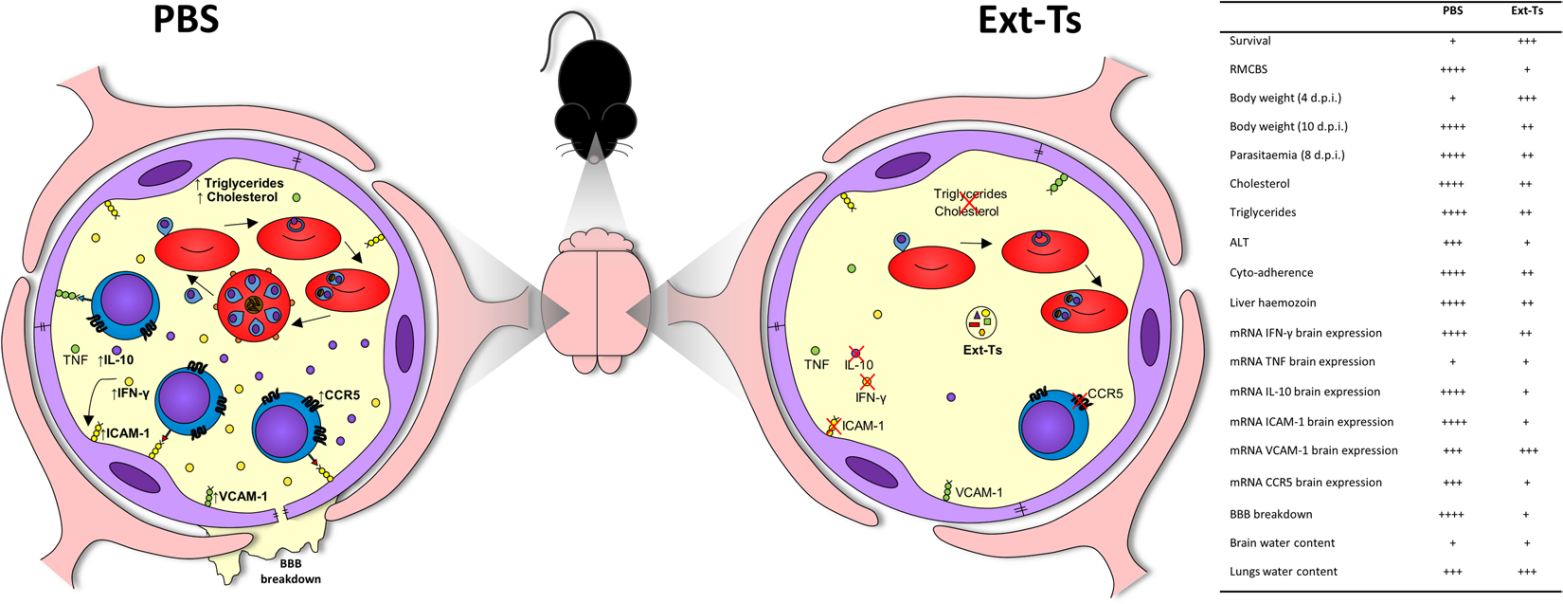
Schematic representation of protective mechanism against ECM induced by Ext-Ts-treatment on PbA-infected mice. Ext-Ts-treatment decreases the triglycerides and cholesterol systemically delaying the parasite development. In the brain the Ext-Ts-treatment decreases the IFN-γ, ICAM-1, VCAM-1 and CCR5 mRNA expression suggesting a decrease in these protein expressions, protecting the integrity of microvasculature. Therefore, the Ext-Ts inhibits the cyto-adherence, obstruction of the cerebral blood vessels and blood-brain barrier disruption. The reduction of cyto-adherence allows the parasite clearance. YC has drawn the figure.

Thus, the lower intensity of cerebral lesions observed in Ext-Ts-treated PbA-infected mice in our investigation was related to the lower IFN-γ and consequently lower ICAM-1 and VCAM-1 expression in the organ as well as the decrease of chemokine receptor CCR5 involved in leukocyte trafficking. Taken together, our results indicate that the Ext-Ts treatment decreases serum lipids, limiting the growth of the PbA parasite in the initial phase of infection and consequently reducing the expression of IFN-γ, ICAM-1, CCR5 and (despite not statistically significant) VCAM-1 in the brain, preserving blood brain barrier integrity and avoiding ECM development (Fig. 9).

When we tested whether the beneficial effect of Ext-Ts treatment was due to general growth inhibition exerted by yet unidentified components of the extract, we observed that ExT-Ts is able to inhibit the *P*. *falciparum* growth in concentrations above 3 μg/mL. *In vivo*, it was detected an inhibition of mouse parasitaemia until 7 d.p.i. that could be either mediated by metabolites of Ext-Ts after injection into mice, or the observed effect is solely mediated by the modified immune reaction of the mice.

Taken together, these results suggest that isolated components of *T*. *stromaticum* could be good anti-malarial drug candidates.

## Materials and Methods

### *Plasmodium falciparum* NF54 cell culture and growth inhibition assays

Growth inhibition assays were conducted in triplicates in 96well flat-bottom culture plates in humidified candle jars to avoid evaporation. Using a stock solution of Ext-Ts at 2 mg/ml in PBS, this stock was serially diluted starting from 50 µg/ml to 0.33 µg/ml in complete RPMI medium supplemented with 0.5% Albumax 1. In parallel, a control culture triplicate was treated with the maximum amount of PBS contained in the 50 µg/ml sample. The starting parasitemia was 0.5% and parasites were grown without medium change for 48 h after which aliquots of IRBCs were analyzed by cytometry in a Guava easycyte apparatus, detecting parasites by Ethidium bromide staining as described previously^62^. The gating settings are documented in Supplementary Informations. The morphology of parasites after treatment was monitored by Giemsa-stained blood smears.

### Maintenance of *T*. *stromaticum* cultures and crude ethanolic extract preparation (Ext-Ts)

The ALF 64 strain of the fungus *Trichoderma stromaticum*, used in the preparation of Tricovab® (Cepec-Ceplac; Centro de Pesquisas do Cacau - Comissão Executiva do Plano da Lavoura Cacaueira), was used in this study and was kindly provided by Dr. Jane Lima dos Santos (State University of Santa Cruz, UESC, Ilhéus- BA). *T*. *stromaticum* was cultivated on potato dextrose agar (PDA) in Petri plates at 25 °C in the dark until observation of conidia production (7–15 days).

After *T*. *stromaticum* sporulation in plates containing PDA, the cultures were washed with 95% ethanol. The ethanolic solution was homogenised in a shaker for 24 hours, after which it was centrifuged at 2200 × g for 20 minutes. The supernatant was collected and dried under a vacuum; the obtained crude extract was weighed, resuspended in sterile phosphate buffered saline (PBS) and stored at −20 °C until use^63^.

### Parasite strain

A PbA strain expressing green fluorescent protein (GFP) was kindly provided by Dr. Ricardo T. Gazzinelli and was used to infect the animals in this study. The strain was kept frozen in liquid nitrogen and thawed to subsequently perform two passages by intraperitoneal (i.p.) inoculation in C57BL/6 mice. Tail blood was collected from a mouse with RBCs containing mainly parasites in ring stage to infect experimental animals.

### Experimental animals and ethical approval

Male C57BL/6 mice at age of 8 to 12 weeks were housed in specific-pathogen-free conditions and received water and food *ad libitum*. Animal experiments were conducted according to institutional guidelines for animal ethics and were approved by the Animal Experimental Ethics Committee (CEUA) of the Federal University of Uberlândia under protocol number 095/15.

### Experimental Procedure

Mice were intraperitoneally inoculated with 5 × 10^4^ RBCs infected with PbA. Four hours after infection^64^ the first dose of Ext-Ts diluted in 200 μL PBS, was inoculated i.p. Mice were treated with 50 mg/Kg/day of Ext-Ts for 10 days, 100 mg/Kg/day of Ext-Ts for 10 or 18 days or 200 mg/Kg/day of Ext-Ts for 10 days. Control mice were treated i.p. with 100 mg/Kg/day of Ext-Ts for 18 days or only PBS daily.

Survival, weight percentage loss and clinical signs were evaluated daily. To assess specific clinical signs of cerebral malaria, animals were evaluated according to the rapid murine coma and behaviour scale (RMCBS) starting from day 4 post-infection (p.i.). The RMCBS is a quantitative scale that evaluates ten parameters (gait, balance, motor performance, body position, limb strength, touch escape, pinna reflex, toe pinch, aggression and grooming) and is scored from 0 to 20, with a 0 score correlating with the lowest function and a 20 score with the highest, 20 being the maximal score^65^.

Haematocrit was evaluated on day 7 p.i. For this purpose, tail blood samples were collected into capillary heparinized tubes and centrifuged for 10 minutes in a microhaematocrit centrifuge (SISLAB- Tecnologia Laboratorial Ltda- Brazil). After centrifugation, values were obtained by comparison with a standard microhaematocrit scale, and results were expressed as a percentage.

For parasitaemia determination, a drop of tail blood was collected in 500 μL of PBS, and samples were fixed with formaldehyde 4%/0.0075% glutaraldehyde in PBS and stored at 4 °C until processing. Parasitaemia of infected RBCs (iRBCs) was assessed daily during the first 10 days and then every two days, detecting GFP^+^ iRBCs by flow cytometry [FACSCanto II flow cytometer (BD Biosciences, San Jose, CA, USA)]. A total of 30,000 gated events were acquired and recorded for analysis from each sample. In addition, parasitaemia was also evaluated in stained thin blood smears (Instant Prov Rapid haematological staining- NewProv, Brazil) by light microscopy.

After 7 days of infection mice were injected with anaesthetics Ketamine (Syntec Brazil Ltda, SP, Brazil)/Xylazine (Schering-Plough Coopers, SP, Brazil) by i.p. route, blood samples were collected from the retro-orbital plexus and animals were euthanized by cervical dislocation. Serum samples were stored at −80 °C for lipid and cytokine analysis. The brain, liver and lungs were collected and fixed in 10% buffered formalin, and processed routinely for paraffin embedding and sectioning. Tissue sections (4 μm) were stained with Haematoxylin and Eosin (H&E) for histological analyses.

### Assessment of blood-brain barrier permeability

A 2% Evans blue dye diluted in PBS was prepared and 200 μL of the solution was injected i.p. on day 7 p.i. The dye was allowed to circulate for 2 hours, then mice were anaesthetized as previously described and perfused through the heart with 0.9% saline. The brain was collected, weighed and homogenized in 0.5% Na_2_SO_4_ (0.6 mL). Acetone (1.4 mL) was added to extract the dye overnight in capped tubes. These samples were centrifuged at 1000 × g, absorbance was measured by spectrophotometer (Versa Max Microplate reader, Molecular Devices, Sunnyvale, CA, USA) at 620 nm and blue stain was quantified according to a standard curve. The results were expressed as μg of Evans Blue stain/g of tissue^66^.

### Pulmonary and cerebral oedema assessment

The lung and brain water content was evaluated to measure cerebral or pulmonary oedema and was estimated by using the wet-dry method. Briefly, mice were deep anaesthetized and exsanguinated. The brain and lungs were collected on day 7 p.i. and immediately the wet weight was measured. After overnight incubation at 80 °C, the dry weight was obtained. Then, the percent of water content was calculated using the following formula: % of water content = [(wet weight − dry weight)/wet weight] × 100^67^.

### Analysis of hepatic deposition of haemozoin

H&E stained tissue sections of liver were used for quantitative analysis of haemozoin (Hz) accumulation in this organ. Images were taken using a 20 × objective of a Nikon Eclipse Ti-5 microscope. A total of 10 microscopic fields of each tissue sections from each mouse were analysed using a polarized filter to detect depolarization caused by Hz. Two images of each microscopic field were obtained; the first one was captured using the adapted polarized light filter, whereas the second one was captured using conventional microscopy. Using ImageJ software version 1.50i, each image was converted to 8 bit, which converts colour images to grayscale images. The threshold was adjusted in such way that the pixels covered by the haemozoin were coloured red. These areas were measured and expressed as a percentage of the total area (total number of pixels)^68^.

### Measurement of lipids and ALT in serum samples

The serum lipid and ALT levels was determined using commercial kits (Labtest Diagnóstica S.A. - Belo Horizonte, MG, Brazil). Blood samples were centrifuged at 800 × g for 10 minutes and serum aliquots were transferred to a clean tube. ALT, total cholesterol, HDL and triglycerides concentrations in serum were measured following the kit instructions for each parameter. LDL and VLDL serum levels were estimated by using of Friedewald equation^69^.

### Cytokine measurement

Serum concentrations of cytokines were measured by sandwich enzyme-linked immunosorbent assay (ELISA.) The IL-10 (OpTEIA, BD Bioscience, San Diego, CA, USA) and IFN-γ (Duoset R&D Systems, Minneapolis, MN, USA) cytokines were assayed according to instructions from the manufacturers. The concentrations of cytokines in serum samples were calculated from a standard curve of each murine recombinant cytokine. The limit of detection in the ELISAs was 31.3 pg/mL (IFN-γ) and 31.3 pg/mL (IL-10).

### RNA extraction and real time quantitative PCR (qPCR)

Frozen pieces of brain were pulverized into powder in liquid nitrogen. Then, RNA was extracted by the Trizol (Life Technologies, Carlsbad, CA, USA) method and quantified by measuring the absorbance at 260 nm on a GeneQuant spectrophotometer (GE Healthcare). Samples were DNAse treated and transcribed into complementary DNA (cDNA). This reaction was performed using 1000 ng RNA and reverse transcriptase ImProm-II™ (Promega, Madison, WI, USA) on Arktik thermocycler (Thermo Scientific). The cDNA was amplified in an ABI PRISM-7500 sequence detection system (Applied Biosystems – USA) using SYBR Green (Invitrogen, Eugene, Oregon, USA) and gene-specific primers: ICAM-1 Fw 5′-TCGTGATGGCAGCCTCTTATG-3′, Rv 5′-TTTTATGGCCTCCTCCTGAGC-3′; VCAM-1 Fw 5′-GTTTGCAGTCTCTCAAGCTTTT-3′, Rv 5′-CCGATTTGAGCAATCGTTT-3′; CCR5 Fw 5′-GACGTGTCTCACCGCTGG-3′, Rv 5′-TGGTTCTTCCCTGTTGGCA-3′; iNOS Fw 5′-CGAAACGCTTCACTTCCAA-3′, Rv 5′- TGAGCCTATATTGCTGTGGCT-3′; IFN-γ Fw 5′-GGCTGTTTCTGGCTGTTACTGC -3′, Rv 5′-CATCCTTTTGCCAGTTCCTCC-3′; TNF Fw 5′-CCACCACGCTCTTCTGTCTACTG-3′, Rv 5′-GATCTGAGTGTGAGGGTCTGGG-3′; IL-10 Fw 5′-TTTAAGGGTTACTTGGGTTGCC-3′, Rv 5′-CGCATCCTGAGGGTCTTCA-3′; GAPDH Fw 5′-GGAGAAACCTGCCAAGTA TGATG-3′, Rv 5′-CAGTGTAGCCCAAGATGCCC-3′. Ct data (cycle threshold) were normalised to the expression of reference gene (GAPDH) and analysed using 2^−ΔΔCt^ method where ΔΔCt = ΔCt sample − ΔCt control sample, in which ΔCt = Ct (studied gene) − Ct (reference gene).

### Statistical analysis

The Kaplan-Meier method was used to compare the survival rates of the experimental groups and the percent survival was compared using Chi-square test. RMCBS score, body weight and parasitaemia were analysed by Two-way ANOVA followed by Bonferroni’s post-test. Haematocrit, Evans blue dye quantification, brain and lungs water content, mRNA quantification, ALT and lipid levels were compared by using One-way ANOVA followed by Bonferroni’s post-test. Hz deposition was compared by Student’s t-test. Serum cytokine concentrations and *P*. *falciparum* parasite growth were compared using Kruskal-Wallis test following by Dunn’s post-test. Statistical analysis and graphs were performed using GraphPad prism version 5.0 (GraphPad Software, San Diego, CA, USA). Values of p < 0.05 were considered statistically significant.

### Data availability statement

All data generated or analysed during this study are included in this published article (and its Supplementary Informations files).

## Electronic supplementary material

Supplementary Information
