# Supplementary material for: Ethanolic extract of the fungus Trichoderma stromaticum decreases inflammation and ameliorates experimental cerebral malaria in C57BL/6 mice
Source: Sci Rep. 2018 Jan 24;8:1547. doi: 10.1038/s41598-018-19840-x (PMC5784021; doi:10.1038/s41598-018-19840-x)
Supplement: Supplementary file 1 — Supplementary Information [file 41598_2018_19840_MOESM1_ESM.pdf]

Supplementary Information

Ethanolic extract of the fungus *Trichoderma stromaticum* decreases inflammation and ameliorates experimental cerebral malaria in C57BL/6 mice

Yusmaris Cariaco, Wânia Rezende Lima, Romulo Sousa, Layane Alencar Costa Nascimento, Marisol Pallete Briceño, Wesley Luzetti Fotoran, Gerhard Wunderlich, Jane Lima dos Santos, Neide Maria Silva*.

Corresponding author: Dr. Neide Maria Silva: nmsilva@ufu.br

Gating settings for parasitaemia measurement during *in vitro* growth inhibition assays

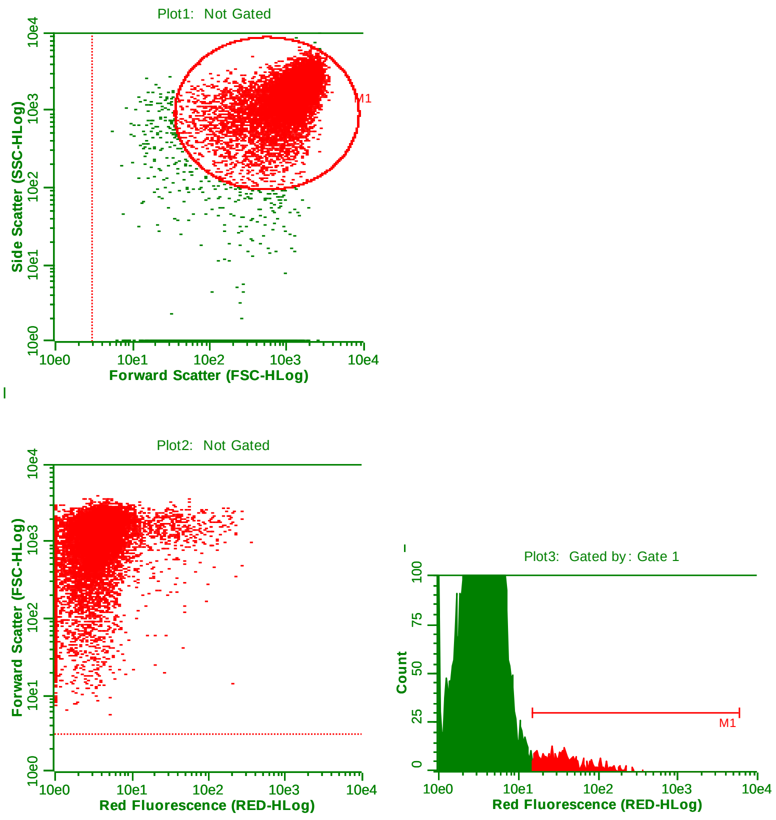

Raw data and gating of untreated and treated NF54 cultures. The upper image shows the selection of cells that were analysed (Gate 1). This gate was applied for all other measurements. The lower picture on the left shows the raw results or forward scatter and red fluorescence (Ethidium bromide-stained nucleated cells, in this case, *P. falciparum*-infected red blood cells). The analysed sample is from the control culture after 48 h growth.

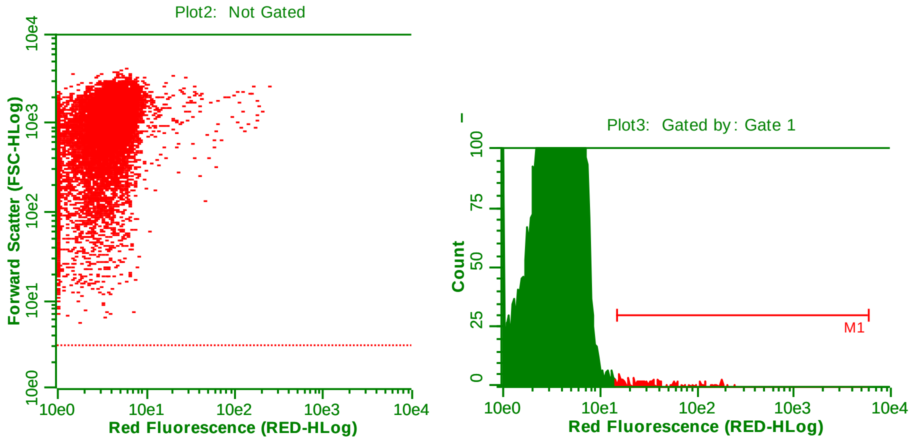

Herein, the raw results for 50 µg/mL-treated parasites are shown in a similar way as above (48 h treatment).

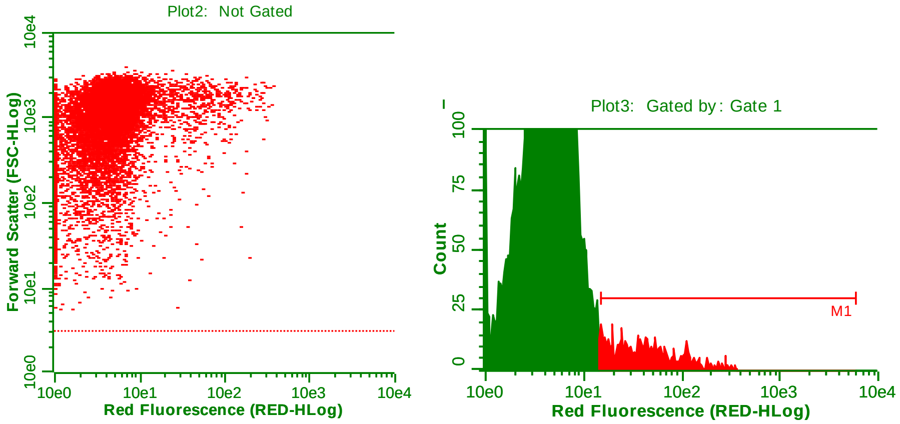

Here, the raw results for 0.75 µg/mL-treated parasites are shown in a similar way as above (48 h treatment).

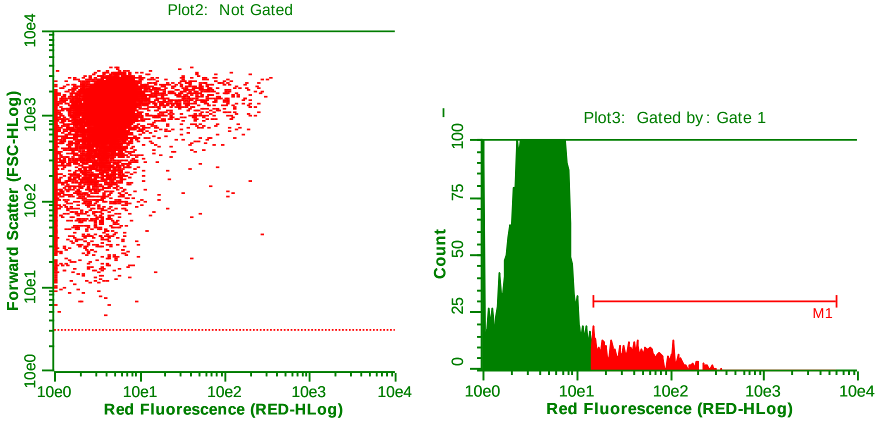

Herein, the raw results for 3 µg/mL-treated parasites are shown in a similar way as above (48 h treatment).
